# Knowledge mapping and visualization analysis of pelvic organ prolapse repair with mesh from 2001 to 2021

**DOI:** 10.3389/fbioe.2023.1104724

**Published:** 2023-04-05

**Authors:** Quan Zhou, Man Lu, Guo-Sheng Li, Gan-Lu Peng, Yan-Feng Song

**Affiliations:** ^1^ Department of Gynecology and Obstetrics, The People’s Hospital of China Three Gorges University/The First People’s Hospital of Yichang, Yichang, China; ^2^ Department of Gynecology and Obstetrics, The 900th Hospital of Joint Logistic Support Force, Fuzhou, Fujian, China

**Keywords:** pelvic organ prolapse, mesh, bibliometric analysis, citation, hotspots

## Abstract

**Aims:** In recent decades, extensive attention has been paid to the application of mesh to repair pelvic floor defects. However, a large body of related literature has not been system summarized. The purpose of this study is to summarize and visualize the literature on pelvic organ prolapse (POP) repair with mesh using bibliometrics.

**Methods:** Medical literature regarding POP repair with mesh were searched and obtained in the Web of Science™ Core (WoSCC) database from 2001 to 2021. Microsoft Excel 2020, CiteSpace and VOSviewer were used to conduct the bibliometric and knowledge-map analysis.

**Results:** In the past 20 years, a total of 2,550 articles and reviews have been published in 35 journals, and the published and cited results show a growing trend. Cosson M and International Urogynecology Journal were the authors and journals with the highest output, respectively. The United States, France and the United Kingdom are among the top three countries/organizations in relevant publications in worldwide. 584 key words in the literature are divided into 8 clusters, which are mainly related to prolapse type, risk factors, surgical methods, imaging, quality of life and bioengineering. Using clinical research and tissue engineering technology to reduce mesh complications is the current hot spot in this field.

**Conclusion:** Reasonable application of mesh and avoiding mesh complications are still the most concerned topics in POP research. Although clinical research, surgical improvement, biological mesh and bioengineering technology have shown promising results, it is still urgent to carry out clinical transformation application research.

## 1 Introduction

Pelvic organ prolapse (POP) is one of the most common chronic medical condition, which attracts wide attention like diabetes, hypertension and coronary heart disease (Barber and Maher, 2013; Pang et al., 2021). Epidemiological studies have found that the incidence rate of POP in postmenopausal women is as high as 50% (Richardson et al., 2009; Barber and Maher, 2013). Most patients with POP have vaginal foreign body sensation and bulge symptom, and severe POP will affects the patient’s urination function, defecation function, and even the quality of sexual life (Mattsson et al., 2020; Raju and Linder, 2021). Conservative treatment, such as pelvic floor muscle exercise, biofeedback therapy and pelvic floor electrical stimulation, has limited therapeutic value for severe POP, pelvic floor repair surgery is the mainstay of treatment for severe POP and is indicated once conservative measures have failed to fully alleviate the symptoms (Hagen and Stark, 2011; Li et al., 2016; Wallace et al., 2019). POP surgery involves multiple organs and disciplines, and there is no unified standard, resulting in a variety of surgical methods (Mattsson et al., 2020; Geoffrion and Larouche, 2021). Up to now, more than 100 surgical procedures have been developed to treat POP (Maher et al., 2019). Therefore, the focus of POP surgery is to develop an efficient, safe, standardized and personalized surgical program.

In the past, traditional operations, such as anterior or posterior plication (vaginoplasty), uterosacral ligament suspension (USLS) or sacrospinal ligaent fixation (SSLF), mainly repair pelvic floor defects by folding, sewing and strengthening their native tissue (Murphy et al., 2021; Yadav et al., 2021). Several studies report that the recurrence rate of traditional surgery is between 17% and 20% 10 years later, which increases the need for further additional treatment and even re-operation (Lowenstein et al., 2018; Cola et al., 2022). In order to overcome the recurrence rate of traditional pelvic floor surgery, gynaecologists tried to implant mesh in the abdomen for POP repair in the 1970s, referring to the hernia mesh repair operation (Zhu and Zhang, 2014; Fleischer and Thiagamoorthy, 2020). Subsequently, these meshes began to be implanted through vagina for POP repair in the 1990s (Dallenbach, 2015; Ugianskiene et al., 2019). In 2002 the FDA approved Gynemesh^®^ PS, which was the first pre-configured transvaginal mesh for surgical repair of POP (Adhoute et al., 2004; Darrow et al., 2021). Since then, due to the excellent anatomical success rate, the application of synthetic mesh in urological surgery has become rapidly popular, leading to a large number of relevant medical devices on the market before obtaining complete clinical trial data (van Geelen and Dwyer, 2013; Heneghan et al., 2017). With the increasing use of synthetic mesh, mesh complications have aroused widespread controversy and concern (Ellington and Richter, 2013). In 2008 and 2011, the FDA of the United States issued safety communication on the transvaginal surgical mesh (TVM) repair for two consecutive times (Winkelman et al., 2019). In 2016, the FDA finalized the devices of TVM for the treatment of prolapse was reclassified as Class III (high-risk) equipment (Food and Drug Administration HHS, 2016). In April of 2019, the FDA banned the sale and distribution of all TVM for POP repair in the United States. Subsequently, the United Kingdom, Canada, Australia, New Zealand and other countries issued bans on TVM (Fairclough et al., 2020; Ng-Stollmann et al., 2020; Shoureshi et al., 2021). However, trans-abdominal mesh pelvic floor repair and TVM surgery in most mainland European countries, Asia, and South America still available as a surgical option for POP correction (Ng-Stollmann et al., 2020). How to use pelvic floor mesh is still a key and puzzling problem in pelvic floor surgery. In addition, tissue engineering technology, new materials and new technologies have been continuously applied in pelvic floor reconstruction surgery, which has expanded the application scope of mesh in pelvic floor defect repair (Yang et al., 2021). However, in the last 2 decades, a number of academic literature on pelvic floor mesh surgery have not been well summarized and discussed. It is necessary to comprehensively analyze the current application trend, progress and hot spot direction of POP repair with mesh.

Bibliometrics is a popular tool for the qualitative and quantitative evaluation of publications in specific fields using mathematical and statistical methods (Ninkov et al., 2022). At present, as a new and promising method, it has been widely used in many biomedical and material fields (Cooper, 2015; Tarnok, 2021). Unfortunately, there is no bibliometric analysis that focused on pelvic floor mesh surgery. In our study, we aim to systematically summarize and sort out the “pelvic organ prolapse repair with mesh” over the last 2 decades by bibliometric analysis. This study combines bibliometrics, mathematics, statistical methods and data visualization to conduct systematic annual, national/regional, institutional, journal/co-cited journals, co-rated reference, timeline view, and the keyword co-occurrence and citation bursts analysis, so as to determine the research trend and hotspot of pelvic floor mesh surgery. It was hoped that this study would provide new interesting insights for the subsequent study of pelvic floor mesh surgery.

## 2 Materials and methods

### 2.1 Articles retrieval

The associated data was retrieved and downloaded from WoSCC on 14 October 2022. The retrieval formula of POP was “Pelvic Organ Prolapse" [Mesh] OR “Cystocele” OR “Rectal Prolapse” OR “Uterine Prolapse” OR “Vaginal prolapse” OR “anterior vaginal wall prolapse” OR “anterior vaginal wall prolapse” OR “posterior vaginal wall prolapse” OR “Vaginal fornix prolapse” OR “uterine stump prolapse”. The retrieval formula of mesh was “Surgical Mesh” [Mesh] OR “transvaginal mesh” OR “grafts” OR “implants”. The retrieval time range was from 1 January 2001 to 31 December 2021, and the search was performed in all language and document types. The retrieved articles were exported in the form of “Full Record and Cited References” and saved in “Plain Text”.

### 2.2 Data analysis

**FIGURE 1:**
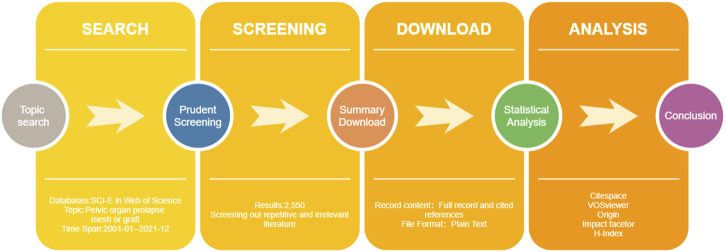
Flow chart of scientific and bibliometric analysis. SCI-E = Science citation index expand; WOS = Web of science; IF = impact factor; H-index = high citation index.

In this study, Microsoft Office Excel 2020 (Microsoft Corporation, Redmond, WA, United States) was used to save and manage relevant data and we used the Origin 2019b software (OriginLab, Northampton, MA, United States) to analyze and plot annual publication output. CiteSpace [version 5.8.R3 (64-bit), Drexel University, United States] was initially used for bibliometric analysis, including country, institute, keyword, category, reference, and cited journal (Chen, 2004; Chen et al., 2014). The time slice (2001–2021), node type and selection criteria (the top 50 levels with the highest number of references or occurrences) of each slice were generally set. In addition, VOSviewer [version 1.6.6, Leiden University, Netherlands] was used to optimize and visualizes scientific knowledge map (van Eck and Waltman, 2010). We also add the impact factor (IF) and H index to the data table through query for comprehensive analysis of scientific measurement results (Hirsch, 2005). In order to avoid missing data, the two individuals carried out all the search and analysis processes. The process of literature retrieval and scientific metrological research is shown in Figure 1.

## 3 Results

### 3.1 Overview, publication outputs and citation trend

**FIGURE 2:**
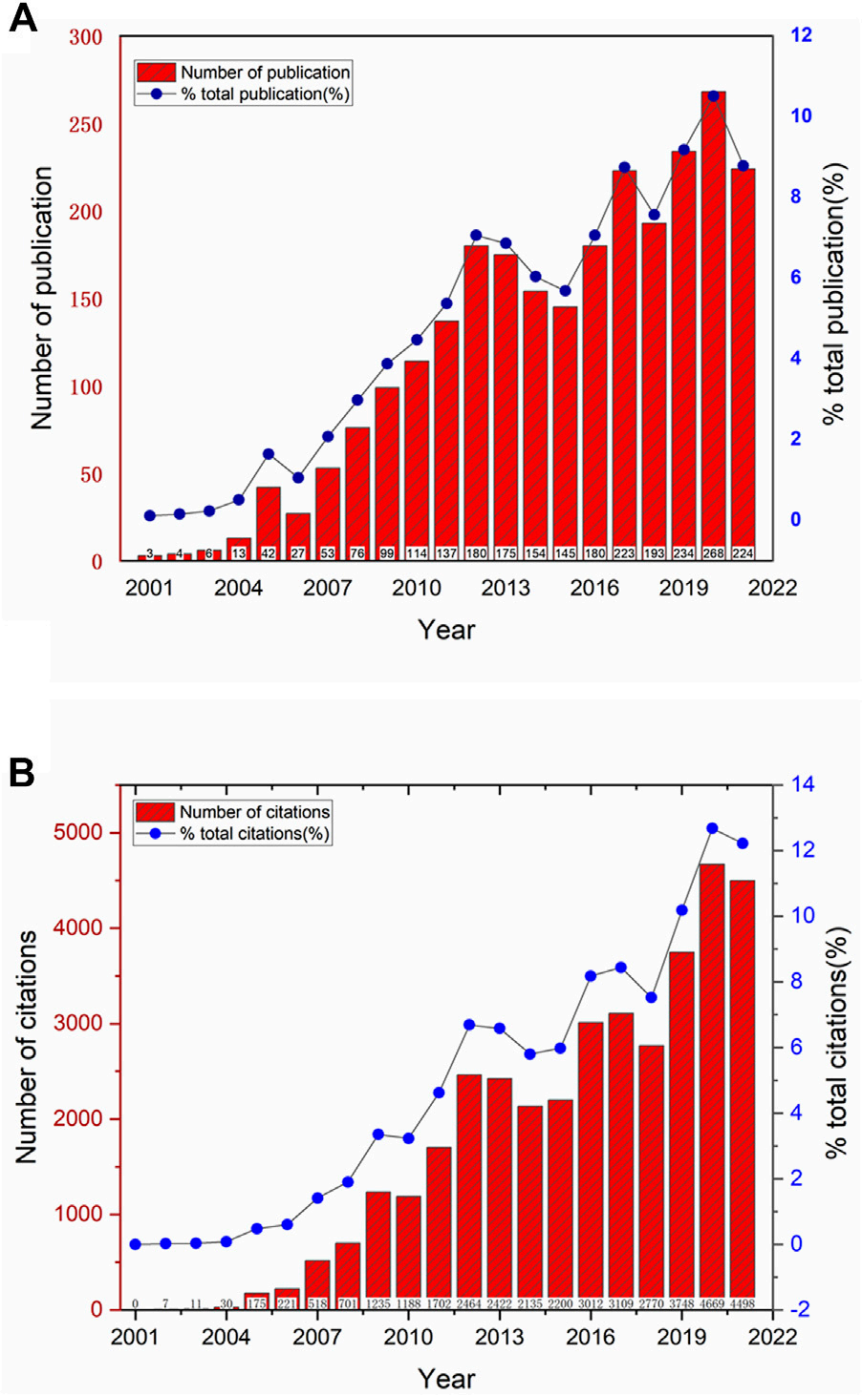
The annual publication and annual citation trends related to POP repair with mesh in the past 20 years. **(A)** The red bars represents the annual publication per year, the blue line represents the trend of the proportion of annual publication in the total number of publications, and the specific proportion (%) of the annual publication in the total number of publications is indicated by the blue solid point. **(B)** The red bars represent the annual citation per year, the blue line represents the trend of the proportion of annual citation in the total number of citations, and the specific proportion (%) of the annual citation in the total number of citations is indicated by the blue solid point.

A total of 2,550 articles related to “pelvic organ prolapse repair with mesh” from the last 2 decades were identified. The number of annual publications was shown in Figure 2A **.** The number of publications has increased from 3 in 2010 to 266 in 2021. Although there were some fluctuations, the overall trend of annual publications was growing. The trend of publications can be divided into four stages: the initial stage (2000–2004), with a slow rate of publications; the rapid growth stage (2005–2012), with accelerating output, the slight falling back stage (2013–2015), with a slight falling trend; and the overall growth stage (2016–2021), with volatile growth, and reached a peak in 2020. The literature is published by multiple publishers, involving multiple subject categories.The number of publishers and categories were shown in Table 1. The top five largest publishers are Springer Nature (753), Elsevier (602), Wiley (368), Lippincott Williams and Wilkins (340) and Taylor and Francis (van Eck and Waltman, 2010). The main research categories involved are Obstetrics Gynecology (1,483), Urology Nephrology (1,145), Surgery (142), General Internal (119), Medicine and Reproducive Biology (110). The number of annual citations was shown in Figure 2B **.** The total number of citations for the retrieved articles was 39,850, and the mean citations per article was 15.6. The H-index of all the selected publications was 82. Correspondingly, the number of annual citations exhibited a similar upward trend, steadily increasing from 0 in 2001 to 4,498 in 2021.

**TABLE 1:** Top 10 publishers and categories related to pelvic organ prolapse repair with mesh in the past 20 years.

Rank	Publishers	Counts	Counts (%)	Rank	Category	Counts	Counts (%)
1	Springer Nature	753	29.529	1	Obstetrics gynecology	1,483	58.157
2	Elsevier	602	23.608	2	Urology nephrology	1,145	44.902
3	Wiley	368	14.431	3	Surgery	142	5.569
4	Lippincott Williams & Wilkins	340	13.333	4	General internal medicine	119	4.667
5	Taylor and Francis	35	1.373	5	Reproductive biology	100	3.922
6	Filodiritto Publisher	24	0.941	6	Engineering	83	3.255
7	Thieme Medical Publishers	24	0.941	7	Materials science	75	2.941
8	Mary Ann Liebert, Inc.	23	0.902	8	Gastroenterology hepatology	46	1.804
9	Brazilian Soc Urol	22	0.863	9	Radiology nuclear medicine medical imaging	33	1.294
10	Karger	21	0.824	10	Science technology other topics	28	1.098

### 3.2 Distribution of country/region and institutions

**FIGURE 3:**
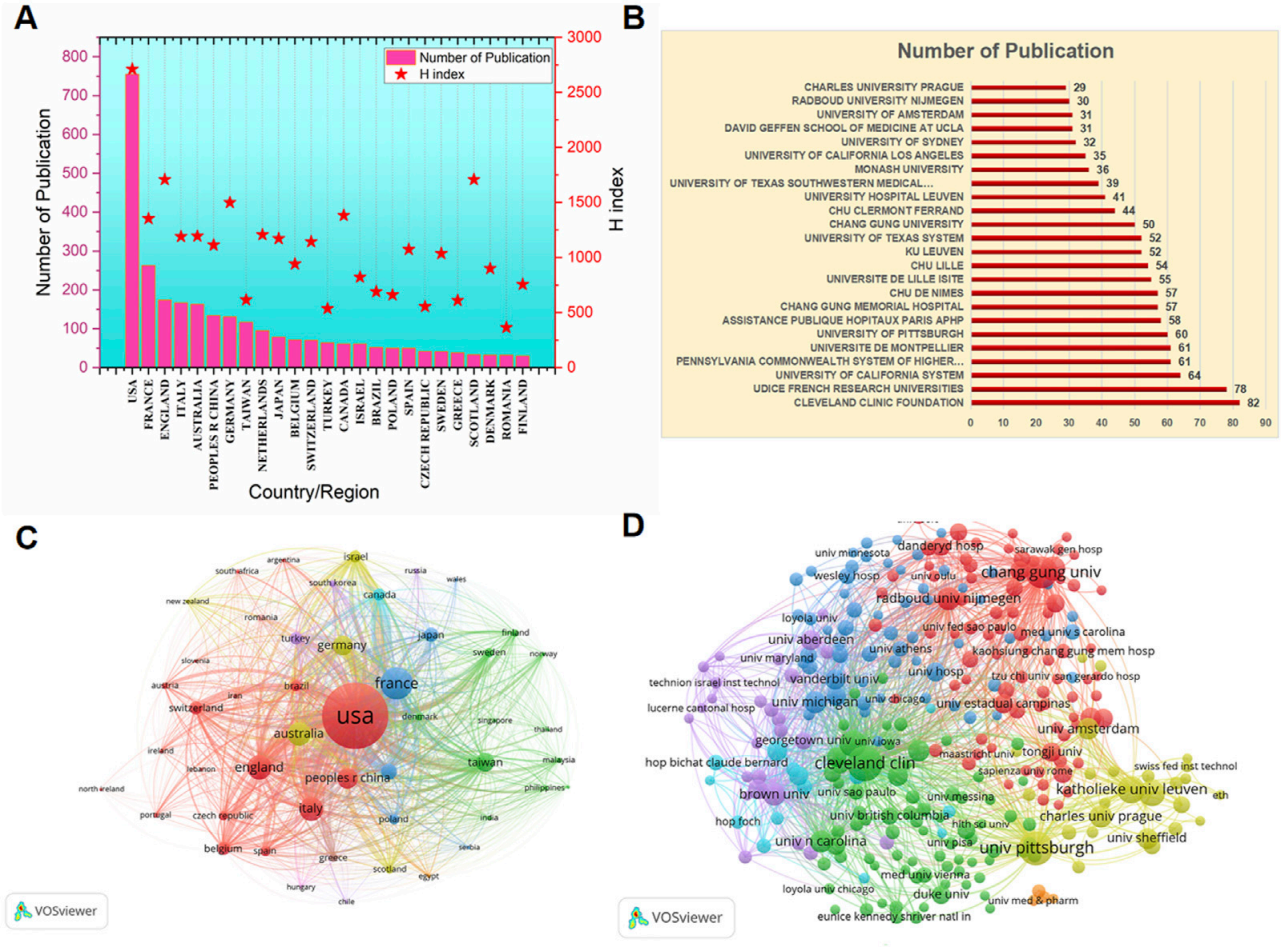
The top most productive of countries/regions and institutions related to POP repair with mesh in the past 20 years. **(A)** The top most productive countries/regions. The red bars represents the number of publications, and the red star represents the H-index of the corresponding country/region. **(B)**The top most productive institutions.The red bars represents the number of publications. **(C)** The cooperation network among the top 30 most productive countries/regions. **(D)** The cooperation network among the top 100 most productive institutions.

All publications covered 65 countries/regions and 2,294 institutions. The top countries/regions in terms of the total number of articles published are illustrated in Table 2; Figure 3A. The United States contributed the most publications (29.61%, with 755 papers), followed by France (10.31%, with 263 papers), England (6.82%, with 174 papers), Italy (6.55%, with 167 papers), Australia (6.39%, with 163 papers), and China (5.26%, with 134 papers). In addition, we further identified the H-index of the top productive countries/regions. The United States (2,711), England (1707), Germany (1,498), Canada (1,381), and France (1,352) has an obvious advantage and makes prominent contributions to mesh research. The top productive institutions were shown in Figure 3B. The leading institutions were Cleveland clinic foundation (3.22%, with 82 papers), UDICE French research universities (3.06%, with 78 papers), university of California system (2.51%, with 64 papers), Pennsylvania commonwealth system of higher education pcshe (2.39%, with 61 papers) and university de montpellier (2.39%, with 61 papers). Most of the high-yield institutions came from Europe and the United States. As shown inFigures 3C, D, the analysis of international cooperation network shows that the United States, with the largest output, cooperates closely. The countries/regions that cooperate most with the United States were England, Italy, Australia, Germany, France and China. In addition, the institutions have also carried out close cooperation, with more research cooperation, and the central positions were Cleveland clinic foundation, University of Pittsburgh, Chang Gung University and University Hospital Leuven.

**TABLE 2:** Top 10 countries and organizations related to pelvic organ prolapse repair with mesh in the past 20 years.

Rank	Country/Region	Counts	Counts (%)	H index	Rank	Organizations	Counts	Counts (%)	Global rank
1	Usa	755	29.608	2,711	1	Cleveland Clinic Foundation	82	3.216	248
2	France	263	10.314	1,352	2	Udice French Research Universities	78	3.059	683
3	England	174	6.824	1707	3	University Of California System	64	2.51	36
4	Italy	167	6.549	1,189	4	Pennsylvania Commonwealth System Of Higher Education Pcshe	61	2.392	32
5	Australia	163	6.392	1,193	5	Universite De Montpellier	61	2.392	258
6	Peoples R China	134	5.255	1,112	6	University Of Pittsburgh	60	2.353	86
7	Germany	131	5.137	1,498	7	Assistance Publique Hopitaux Paris Aphp	58	2.275	75
8	Taiwan	117	4.588	615	8	Chang Gung Memorial Hospital	57	2.235	461
9	Netherlands	95	3.725	1,206	9	Chu De Nimes	57	2.235	685
10	Japan	79	3.098	1,171	10	Universite De Lille Isite	55	2.157	458

### 3.3 Authors and Co-Cited authors

**FIGURE 4:**
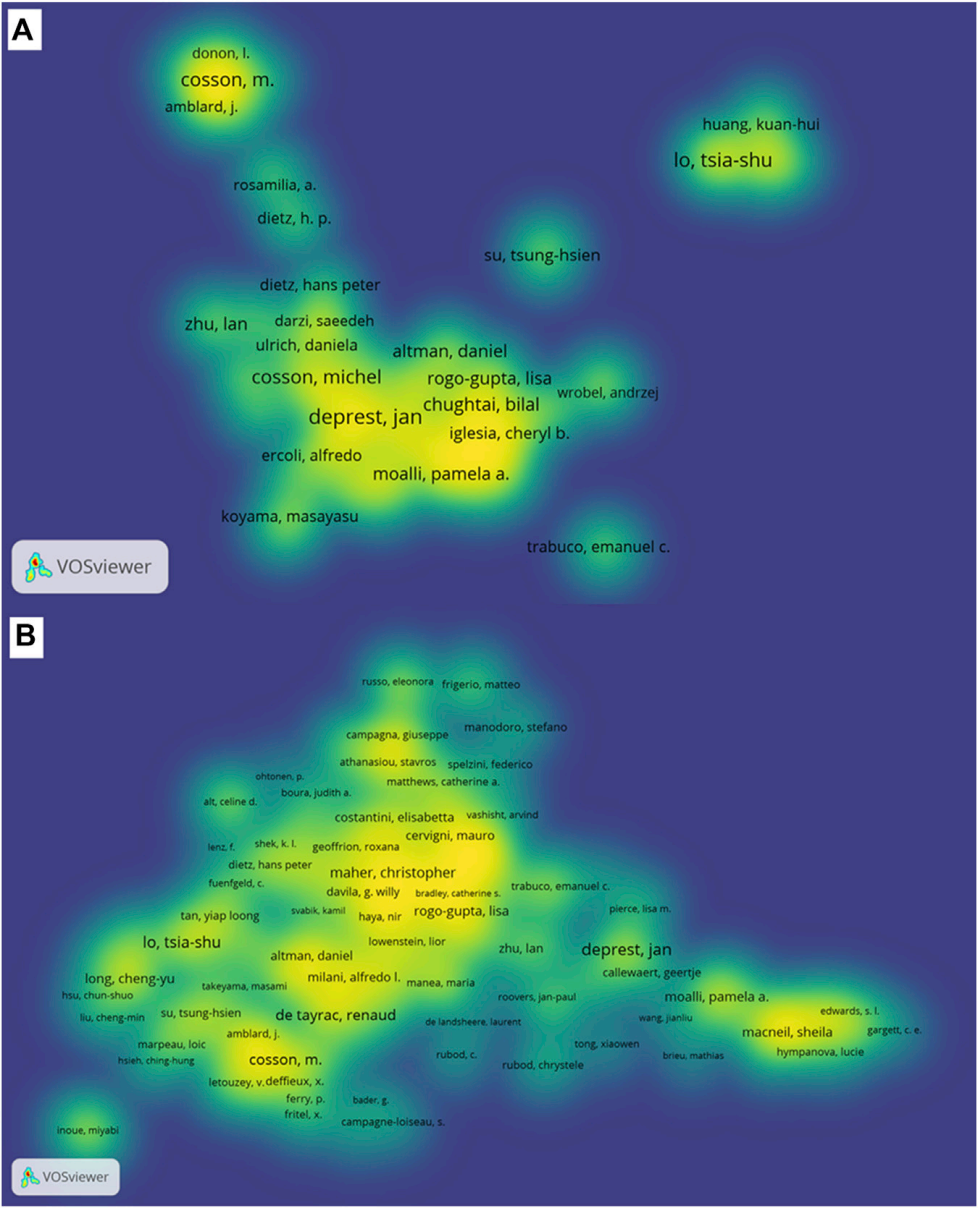
The density map of authors and co-cited authors related to POP repair with mesh in the past 20 years. **(A)** The spectral density map of the top 100 most productive authors. **(B)** The density map of the co-cited authors.In this cluster density map, authors with close relationship are allocated to one cluster with the same color.

A total of 721 authors and 891 co-cited authors participated in publishing the related literature involved in this analysis. The top 100 most productive authors and co-cited authors are displayed in spectral density maps in Figure 4, and the details of the top 10 authors or co-cited authors in both rankings are listed in Table 3. From the Figure 4, we can see the close cooperation between different authors and co-cited authors, which may play an important role as a bridge for in-depth research in this field. As shown in Table 3, the most published papers are Cosson M (*n* = 50), followed by De Tayrac R (*n* = 43), Deprest J (*n* = 42), Maher C (*n* = 32), and Fatton B (*n* = 31). The centrality of the top 10 authors ranges from 0.01–0.14, the highest centrality f Deprest J and Lo T is 0.14, and the lowest centrality of Abramowitch S and Jacquetin B is 0.01. The top five frequently co-cited authors are Maher C (856 Citation), Olsen AL (702 Citation), Bump RC (588 Citation), Altman D (536 Citation) and Barber MD (527 Citation).

**TABLE 3:** Top 10 The top 10 authors and co-cited authors related to pelvic organ prolapse repair with mesh in the past 20 years.

Rank	Author	Counts	Centrality	Rank	Co-cited author	Citation	Centrality
1	Cosson M	50	0.12	1	Maher C	856	0.02
2	De Tayrac R	43	0.04	2	Olsen AL	702	0.02
3	Deprest J	42	0.14	3	Bump RC	588	0.02
4	Maher C	32	0.12	4	Altman D	536	0.04
5	Fatton B	31	0.09	5	Barber MD	527	0.02
6	Moalli P	27	0.05	6	Haylen BT	405	0.01
7	Gargett C	27	0.03	7	De Tayrac R	388	0.04
8	Lo T	24	0.14	8	Weber AM	360	0.02
9	Abramowitch S	23	0.01	9	Maher CF	346	0.05
10	Jacquetin B	21	0.01	10	Wu JM	322	0.02

### 3.4 Journals and co-cited journals

The top 30 most productive journals and the most cited journals are listed in Table 4; Table 5, respectively. International urogynecology journal (21.49%, with 548 papers) published the most papers in this field, followed by Neurourology and urodynamics (4.902%, with 125 papers), Female pelvic medicine and reconstructive surgery (4.824%, with 123 papers), European journal of obstetrics and gynecology and reproductive (3.765%, with 96 papers), American journal of obstetrics and gynecology (3.137%, with 80 papers) and Journal of minimally invasive gynecology (2.235%, with 57 papers). Among the top 30 most prolific journals, European urology had the highest IF (2021) of 24.27, and American journal of obstetrics and gynecology had the highest H-Index (2021) of 235. It was regrettable that 80% were classified as JCR Q3 or Q4. Based on the cited number of publications, the most frequently co-cited journal also was International urogynecology journal (15.89%, with 1,578 total citations), followed by Female pelvic medicine and reconstructive surgery (4.04%, with 401 total citations), Neurourology and urodynamics (3.89%, with 386 total citations), European journal of obstetrics and gynecology and reproductive (2.63%, with 261 total citations) and American journal of obstetrics and gynecology (2.34%, with 225 total citations). Compared with journal analysis, the quality of co-cited journals was better and involves multiple disciplines, which indicates that the application of pelvic floor mesh requires more high-quality evidence and interdisciplinary collaboration.

**TABLE 4:** Top 30 most active journals related to pelvic organ prolapse repair with mesh in the past 20 years.

Ranking	Journal	Counts	% Of counts	Country/Region	JCR partition	Impact factor (2021)	H-Index (2021)
1	International Urogynecology Journal	548	21.49	United Kingdom	Q4	1.93	97
2	Neurourology and Urodynamics	125	4.90	United States	Q3	2.37	95
3	Female Pelvic Medicine and Reconstructive Surgery	123	4.82	United States	Q4	1.91	31
4	European Journal Of Obstetrics Gynecology and Reproductive Biology	96	3.77	Ireland	Q3	2.83	104
5	American Journal Of Obstetrics and Gynecology	80	3.14	United States	Q1	10.69	235
6	Journal Of Minimally Invasive Gynecology	57	2.24	Netherlands	Q1	4.31	84
7	Journal Of Urology	57	2.24	United States	Q1	7.60	265
8	Obstetrics and Gynecology	55	2.16	United States	Q1	7.62	231
9	Bjog An International Journal Of Obstetrics and Gynaecology	50	1.96	United Kingdom	Q1	7.33	170
10	Urology	45	1.77	United States	Q3	2.63	182
11	Progres En Urologie	34	1.33	France	Q4	1.09	34
12	Archives Of Gynecology and Obstetrics	32	1.26	Germany	Q3	2.49	73
13	Current Opinion In Urology	32	1.26	United States	Q3	2.81	123
14	Current Opinion In Obstetrics Gynecology	28	1.10	United States	Q4	2.21	79
15	Current Urology Reports	28	1.10	United States	Q3	2.86	45
16	Journal Of Obstetrics and Gynaecology Research	28	1.10	Australia	Q4	1.70	56
17	International Journal Of Gynecology Obstetrics	27	1.06	United Kingdom	Q1	4.45	103
18	Taiwanese Journal Of Obstetrics Gynecology	24	0.94	Taiwan	Q4	1.94	39
19	International Braz J Urol	22	0.86	Brazil	Q2	3.05	41
20	Australian New Zealand Journal Of Obstetrics Gynaecology	20	0.78	United States	Q4	1.88	68
21	International Journal Of Urology	20	0.78	United Kingdom	Q3	2.90	71
22	Obstetrical Gynecological Survey	20	0.78	United States	Q3	3.01	85
23	Gynecologie Obstetrique Fertilite	18	0.71	France	Q4	1.06	36
24	European Urology	17	0.67	Netherlands	Q1	24.27	230
25	Journal De Gynecologie Obstetrique Et Biologie De La Reproduction	17	0.67	France	Q4	0.77	33
26	Clinical Obstetrics and Gynecology	15	0.59	United States	Q4	1.97	79
27	Ginekologia Polska	15	0.59	Poland	Q4	1.22	24
28	Journal Of Obstetrics And Gynaecology	15	0.59	United Kingdom	Q4	1.23	51
29	Journal Of The Mechanical Behavior Of Biomedical Materials	15	0.59	Netherlands	Q2	4.04	99
30	Acta Obstetricia Et Gynecologica Scandinavica	14	0.55	United Kingdom	Q1	4.54	106

**TABLE 5:** Top 30 most co-cited journals related to pelvic organ prolapse repair with mesh in the past 20 years

Ranking	Journal	Counts	% Of counts	Country/Region	JCR partition	Impact factor (2021)	H- Index (2021)
1	International Urogynecology Journal	1,578	15.89	United Kingdom	Q4	1.93	97
2	Female Pelvic Medicine and Reconstructive Surgery	401	4.04	United States	Q4	1.91	31
3	Neurourology and Urodynamics	386	3.89	United States	Q3	2.37	95
4	European Journal Of Obstetrics Gynecology and Reproductive Biology	261	2.63	Ireland	Q3	2.83	104
5	American Journal Of Obstetrics and Gynecology	235	2.37	United States	Q1	10.69	235
6	Obstetrics and Gynecology	174	1.75	United States	Q1	7.62	231
7	Journal Of Minimally Invasive Gynecology	162	1.63	Netherlands	Q1	4.31	84
8	Urology	126	1.27	United States	Q3	2.63	182
9	Archives Of Gynecology and Obstetrics	119	1.20	Germany	Q3	2.49	73
10	Bjog An International Journal Of Obstetrics and Gynaecology	117	1.18	United Kingdom	Q1	7.33	170
11	Journal Of Urology	115	1.16	United States	Q1	7.60	265
12	Progres En Urologie	100	1.01	France	Q4	1.09	34
13	International Journal Of Gynecology Obstetrics	93	0.94	United Kingdom	Q1	4.45	103
14	Colorectal Disease	86	0.87	United Kingdom	Q2	3.92	95
15	Ultrasound In Obstetrics Gynecology	83	0.84	United Kingdom	Q1	8.68	151
16	European Urology	66	0.66	Netherlands	Q1	24.27	230
17	Techniques In Coloproctology	66	0.66	Italy	Q2	3.70	55
18	Journal Of Obstetrics and Gynaecology Research	65	0.65	Australia	Q4	1.70	56
19	Journal Of The Mechanical Behavior Of Biomedical Materials	63	0.63	Netherlands	Q2	4.04	99
20	Current Opinion In Urology	61	0.61	United States	Q3	2.81	123
21	Australian New Zealand Journal Of Obstetrics Gynaecology	58	0.58	United States	Q4	1.88	68
22	Journal Of Obstetrics and Gynaecology	58	0.58	United Kingdom	Q4	1.23	51
23	Journal Of Sexual Medicine	58	0.58	United States	Q2	3.94	123
24	Taiwanese Journal Of Obstetrics Gynecology	53	0.53	Taiwan	Q4	1.94	39
25	Current Opinion In Obstetrics Gynecology	52	0.52	United States	Q4	2.21	79
26	Acta Obstetricia Et Gynecologica Scandinavica	51	0.51	United Kingdom	Q1	4.54	106
27	Current Urology Reports	51	0.51	United States	Q3	2.86	45
28	Scientific Reports	50	0.50	United Kingdom	Q2	5.00	242
29	Acta Biomaterialia	49	0.49	Netherlands	Q1	10.63	207
30	Journal Of Clinical Medicine	49	0.49	Switzerland	Q2	4.96	75

### 3.5 Co-cited reference and timeline view

**FIGURE 5:**
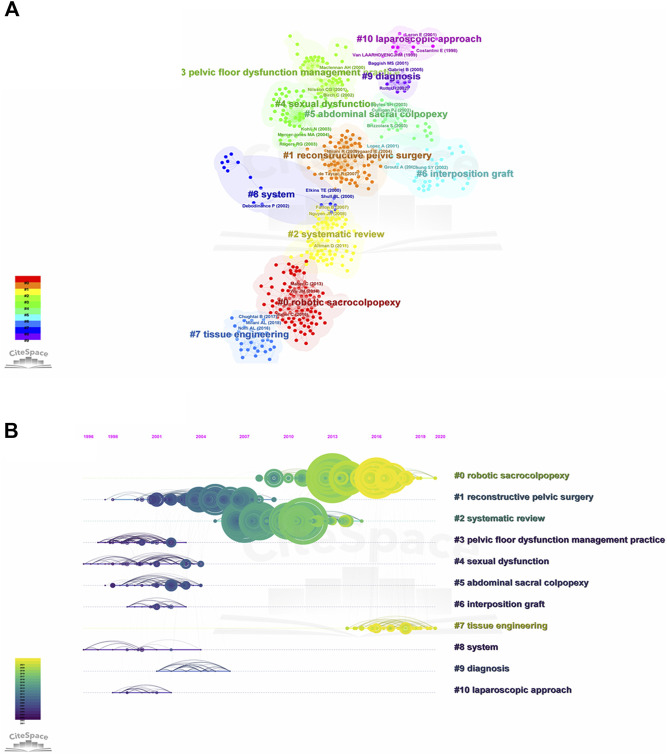
The co-citation visualization network and timeline view of co-cited references related to POP repair with mesh in the past 20 years. **(A)** The co-citation visualization network of co-cited references. Each node represents an article or review, and each frame represents a cluster. The size of each node represent the numbers of co-citations. The labels of the clusters were also displayed adjacent to the frames. **(B)** The timeline view of r co-cited references.The node’s position on the horizontal axis represents the time when the reference first appeared, and the node’s size is positively correlated with the number of citations of the reference. The lines between the nodes represent co-cited relationships. This dark blue means close to 2001, while more yellow means close to 2021.

The top 30 most highly cited references regarding pelvic floor mesh were shown in Table 6. Of them, three references were co-cited over 500 times, four references were co-cited between 300 and 500 times, and others are co-cited between 160 and 300 times. 13 papers were in top journals (IF >10) of the top 30, the top six journals are LANCET (1), New England Journal of Medicine (Barber and Maher, 2013), JAMA (1), European Urology (Barber and Maher, 2013), Cochrane Database of Systematic Reviews (Mattsson et al., 2020), and American Journal of Obstetrics and Gynecology (Mattsson et al., 2020). We have further conducted reference co-citation analysis with CiteSpace. As shown in Figure 5, all included articles were divided into 11 clusters according to their main research topics, including robot sacrocololpopexy (#0), reconstruction pelvic surgery (#1), system review (#2), pelvic floor management practice (#3), sexual dysfunction (#4), abdominal sacrocololpopexy (#5), interposition graft (#6), tissue engineering (#7), system (#8), diagnosis (# 9) and laparoscopic approach (#10). Timeline view analysis shows that reconstruction pelvic surgery (#1), abdominal sacrocololpopexy (#5) and pelvic floor management practice (#3) were relatively early hotspots, while robot sacrocololpopexy (#0) laparoscopic approach (#10), and tissue engineering (#7) are the current research focuses, and the most recent references with citation bursts appeared between 2013 and 2018.

**TABLE 6:** The top 30 most highly cited references related to pelvic organ prolapse repair with mesh in the past 20 years.

Rank	Title	Authors	Source title	Publication year	Total citations	Average per year	If (2021)	H-Index (2021)
1	Abdominal sacrocolpopexy: A comprehensive review	Nygaard, I.E.,	Obstetrics and Gynecology	2004	632	33.26	7.62	231
2	Surgical management of pelvic organ prolapse in women	Maher, Christopher	Cochrane Database of Systematic Reviews	2013	565	56.5	12.01	292
3	Pelvic organ prolapse	Jelovsek, J. Eric	Lancet	2007	528	33	202.73	807
4	Anterior Colporrhaphy versus Transvaginal Mesh for Pelvic-Organ Prolapse	Altman, Daniel	New England Journal of Medicine	2011	415	34.58	176.08	1,079
5	Long-term Outcomes Following Abdominal Sacrocolpopexy for Pelvic Organ Prolapse	Nygaard, Ingrid	Jama-Journal of The American Medical Association	2013	340	34	157.33	709
6	Surgical management of pelvic organ prolapse in women	Maher, Christopher	Cochrane Database of Systematic Reviews	2010	309	23.77	12.01	292
7	Outcome after anterior vaginal prolapse repair - A randomized controlled trial	Nguyen, John N.	Obstetrics and Gynecology	2008	305	20.33	7.62	231
8	Prospective randomized trial of polyglactin 910 mesh to prevent recurrence of cystoceles and rectoceles	Sand, PK	American Journal of Obstetrics and Gynecology	2001	289	13.14	10.69	235
9	Transvaginal repair of genital prolapse: preliminary results of a new tension-free vaginal mesh (Prolift (TM) technique)—a case series multicentric study	Fatton, B.	International Urogynecology Journal	2007	287	17.94	1.93	97
10	Low-weight polypropylene mesh for anterior vaginal wall prolapse—a randomized controlled trial	Hiltunen, Reijo	Obstetrics and Gynecology	2007	240	15	7.62	231
11	Complication and Reoperation Rates After Apical Vaginal Prolapse Surgical Repair A Systematic Review	Diwadkar, Gouri B.	Obstetrics And Gynecology	2009	231	16.5	7.62	231
12	Incidence and management of graft erosion, wound granulation, and dyspareunia following vaginal prolapse repair with graft materials: a systematic review	Abed, Husam	International Urogynecology Journal	2011	227	18.92	1.93	97
13	An International Urogynecological Association (IUGA)/International Continence Society (ICS) joint terminology and classification of the complications related directly to the insertion of prostheses (meshes, implants, tapes) & grafts in female pelvic floor surgery	Haylen, Bernard T.	International Urogynecology Journal	2011	226	18.83	1.93	97
14	Risk factors for pelvic organ prolapse and its recurrence: a systematic review	Vergeldt, Tineke F. M.	International Urogynecology Journal	2015	215	26.88	1.93	97
15	Transvaginal mesh or grafts compared with native tissue repair for vaginal prolapse	Maher, Christopher	Cochrane Database of Systematic Reviews	2016	214	30.57	12.01	292
16	Functional and anatomical outcome of anterior and posterior vaginal prolapse repair with prolene mesh	Milani, R	Bjog-An International Journal of Obstetrics and Gynaecology	2005	214	11.89	7.33	170
17	An International Urogynecological Association (IUGA)/International Continence Society (ICS) Joint Terminology and Classification of the Complications Related Directly to the Insertion of Prostheses (Meshes, Implants, Tapes) and Grafts in Female Pelvic Floor Surgery	Haylen, Bernard T.	Neurourology and Urodynamics	2011	212	17.67	2.37	95
18	The Current Status of Laparoscopic Sacrocolpopexy: A Review	Ganatra, Anjali M.	European Urology	2009	209	14.93	24.27	230
19	Short-Term Outcomes of Robotic Sacrocolpopexy Compared With Abdominal Sacrocolpopexy	Geller, Elizabeth J.	Obstetrics and Gynecology	2008	199	13.27	7.62	231
20	Transvaginal mesh technique for pelvic organ prolapse repair: mesh exposure management and risk factors	Collinet, Pierre	International Urogynecology Journal	2006	190	11.18	1.93	97
21	Surgical management of pelvic organ prolapse in women: A short version Cochrane review	Maher, C.	Neurourology and Urodynamics	2008	185	12.33	2.37	95
22	Surgical management of pelvic organ prolapse in women: the updated summary version Cochrane review	Maher, Christopher M.	International Urogynecology Journal	2011	184	15.33	1.93	97
23	Outcomes after anterior vaginal wall repair with mesh: a randomized, controlled trial with a 3 years follow-up	Nieminen, Kari	American Journal of Obstetrics and Gynecology	2010	184	14.15	10.69	235
24	Efficacy and safety of using mesh or grafts in surgery for anterior and/or posterior vaginal wall prolapse: systematic review and meta-analysis	Jia, X.	Bjog-An International Journal of Obstetrics and Gynaecology	2008	176	11.73	7.33	170
25	Laparoscopic sacral colpopexy versus total vaginal mesh for vaginal vault prolapse: a randomized trial	Maher, Christopher F.	American Journal of Obstetrics and Gynecology	2011	175	14.58	10.69	235
26	Pelvic floor ultrasound: a review	Dietz, Hans Peter	American Journal of Obstetrics and Gynecology	2010	174	13.38	10.69	235
27	Efficacy and safety of transvaginal mesh kits in the treatment of prolapse of the vaginal apex: a systematic review	Feiner, B.	Bjog-An International Journal of Obstetrics and Gynaecology	2009	174	12.43	7.33	170
28	Two-year outcomes after sacrocolpopexy with and without burch to prevent stress urinary incontinence	Brubaker, Linda	Obstetrics and Gynecology	2008	171	11.4	7.62	231
29	Transvaginal repair of anterior and posterior compartment prolapse with Atrium polypropylene mesh	Dwyer, PL	Bjog-An International Journal of Obstetrics And Gynaecology	2004	162	8.53	7.33	170
30	Surgery for women with apical vaginal prolapse	Maher, Christopher	Cochrane Database of Systematic Reviews	2016	167	23.86	12.01	292

### 3.6 Keywords co-occurrence and citation bursts analysis

**FIGURE 6:**
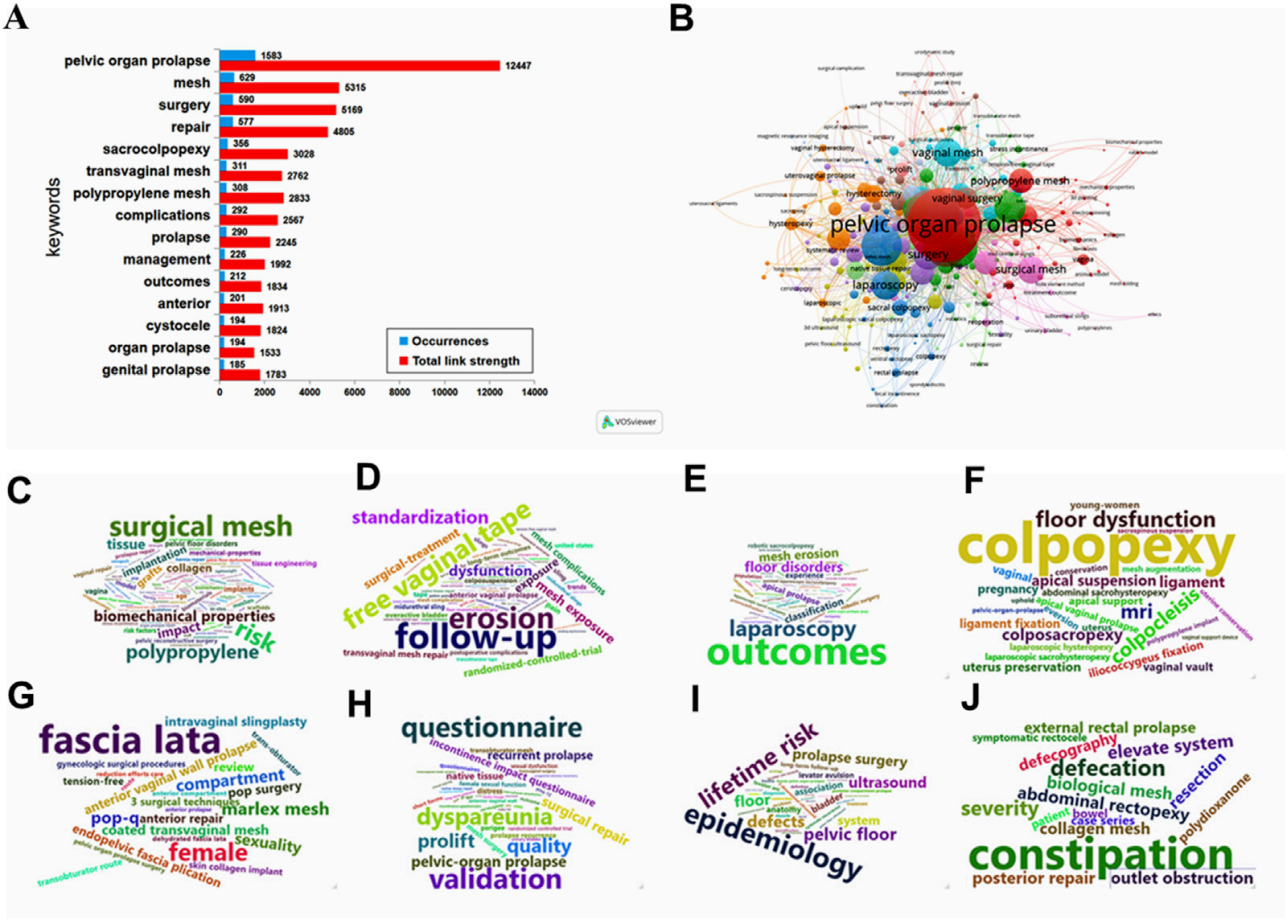
The distribution, co-occurrence network map and word cloud cluster diagram of keywords. **(A)** The distribution of keywords, the blue histograms represent occurrences, and the red histogram represents total link strength. **(B)** The co-occurrence network of keywords, minimum number of occurrences of keywords ≥20. Node size and color represents the number of keywords and cluster. Lines of different colors show that the 2 keywords appear in an article. **(C–J)** The word cloud cluster diagram of mechanism, type of prolapse, mesh complications, minimally invasive surgery, middle pelvic repair, anterior pelvic repair, quality of life, imaging examination, and bioengineering technology, respectively.

**FIGURE 7:**
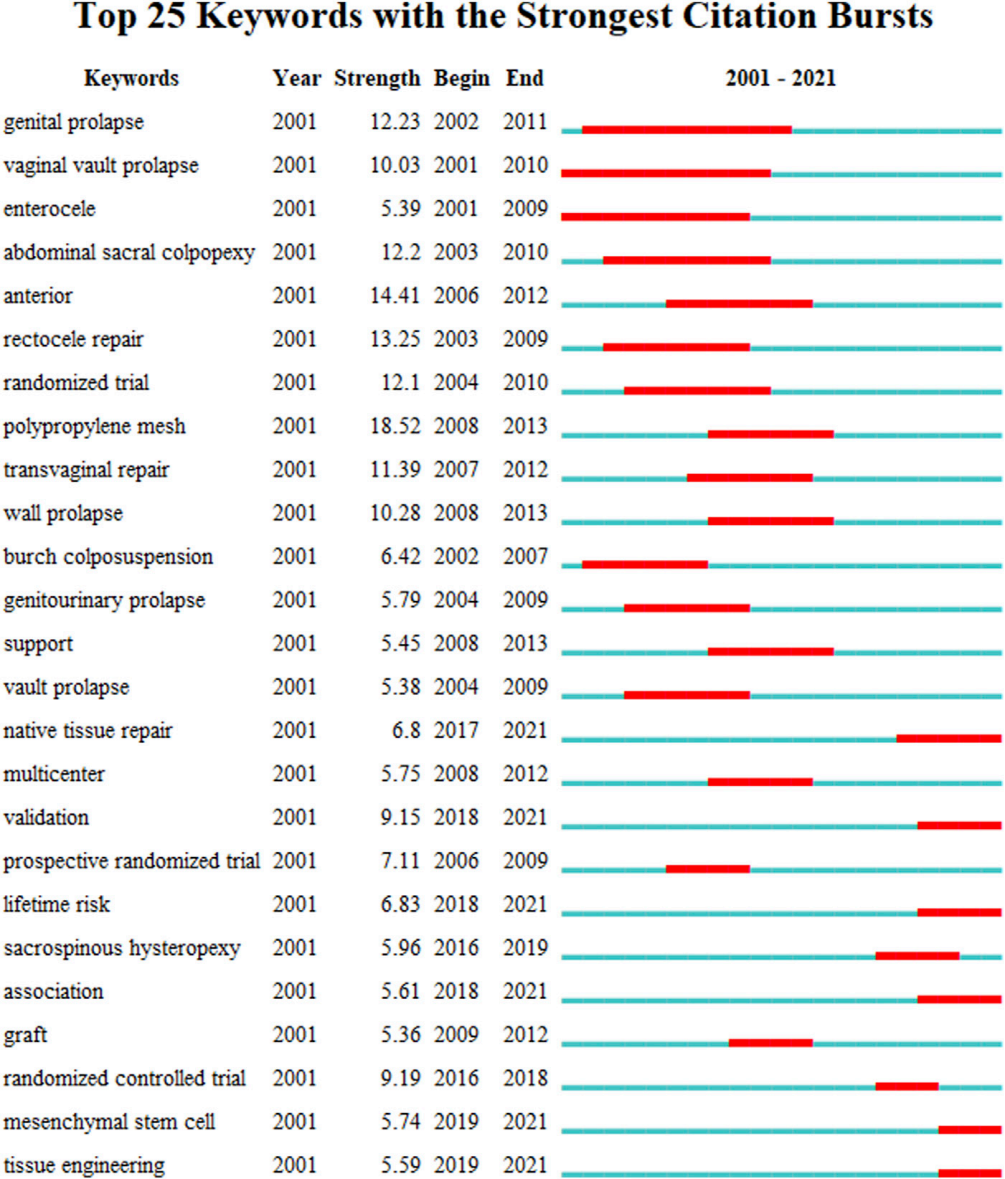
Top 25 keywords with strongest citation bursts. A blue line indicates the timeline, and the intervals in which bursts were found are indicated by red sections on the blue timeline, indicating the start year, the end year, and the burst duration.

To further hot spot analysis, we analyzed the keywords co-ccurrence in the references through VOSviewer. As shown in Figure 6A, the top 20 keywords network with the highest co-occurrence were calculated and displayed by VOSviewer. The most frequently occurring keywords are pelvic organ prolapse (*n* = 1,583), followed by mesh (*n* = 629), surgery (*n* = 590), repair (*n* = 577), women (*n* = 547), sacrocolpopexy (*n* = 356), transvaginal mesh (*n* = 311), polypropylene mesh (*n* = 308), and complications (*n* = 292). These high-frequency keywords, to a certain extent, represent the hot spots in the research on mesh repair of pelvic floor defects. As shown in Figure 6B, the network map of high-frequency keywords can intuitively display. 584 keywords were identified and classified into eight clusters: mechanism, type of prolapse, mesh complications, minimally invasive surgery, middle pelvic repair, anterior pelvic repair, quality of life, imaging examination, and bioengineering technology (Figures 6C-6J). We also used CiteSpace for keywords citation bursts analysis, and the top 30 keywords with the strongest citation bursts were shown in Figure 7. Among them, Keywords related to the concept of disease, such as genital prolapse, vaginal vault prolapse and enterocele, bursts early and were cited intensively. Keywords related to prolapse treatment and clinical research, such as transvaginal repair, polypropylene mesh, graft, support, randomized trial, randomized controlled trial, and multicenter, usually bursts between 2007 and 2009, with moderate stronger citation. More remarkable, we mainly focused on keywords that ongoing burst till 2021, including validation (strength, 9.15; time span, 2018–2021), lifetime risk (6.83, 2018–2021), native tissue repair (6.8, 2017–2021), tissue engineering (5.59, 2019–2021), and mesenchymal stem cells (5.74, 2019–2021). These keywords may be the focus and direction of current pelvic floor medical research.

### 3.7 Artificial clustering analysis of keywords

**FIGURE 8:**
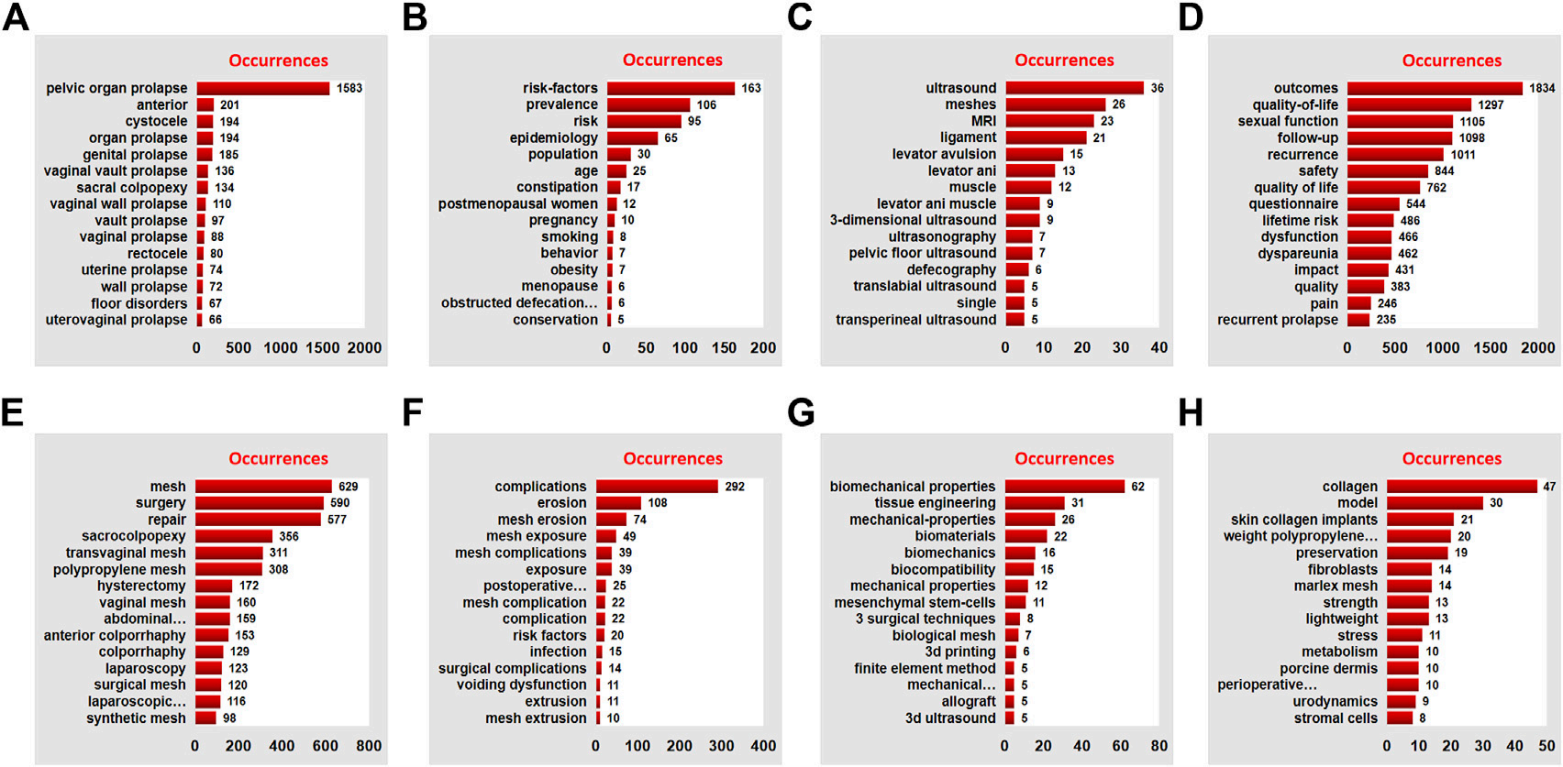
Manual analysis and clustering of keywords. The red histograms represent the occurrences of the keyword: **(A)** Prolapse classification, **(B)** Risk factors, **(C)** Imaging progress, **(D)** Mesh surgery, **(E)** mesh complications, **(F)** quality of life, **(G)** tissue engineering, and **(H)** new research directions, respectively.

In order to further discuss the current research trend of pelvic floor defect mesh repair, based on keyword analysis, we manually classified the keyword network data into eight clusters, including prolapse classification, risk factors, imaging progress, mesh surgery, mesh complications, quality of life, tissue engineering, and new research directions. Through the keywords of cluster 1, we can find that POP is a group of diseases caused by the weakening of pelvic floor supporting tissues, including the prolapse of various organs in the anterior, middle and posterior pelvic cavity, severe POP and some recurrent prolapse requiring surgical treatment (Figure 8A). Key words in cluster 2 show that POP is a multifactorial disease, and related environmental factors can be determined through epidemiological studies, among which aging, obesity, chronic obstructive pulmonary disease (COPD), menopause, pregnancy and childbirth are important risk factors (Figure 8B). Key words in cluster 3 suggest that imaging evaluation, especially ultrasound and MRI, is very important for qualitative and localized diagnosis of pelvic floor muscle, ligament, levator ani hiatus injuries (Figure 8C). In cluster 4, we can speculate that POP has a great impact on the quality of life of patients, so we should pay attention to the evaluation of subjective treatment effect before and after surgery (Figure 8D). For cluster 5, pelvic floor mesh repair is still an effective treatment for some POP patients. There are three ways of implantation: abdominal, vaginal and laparoscopic, and the type of mesh is mainly synthetic polypropylene (Figure 8E). More importantly, the keywords in cluster 6 reflect that the mesh complication is still the most noticeable problem in pelvic floor mesh surgery, especially the exposure, erosion, shrinkage, extrusion and infection of the mesh, which has attracted extensive attention from international experts and academic organizations (Figure 8F). In cluster 7, the research on bioengineering technologies such as mechanical properties, biocompatibility, biomechanics, biomaterials, mechanical properties, tissue engineering, and biological properties has been deeply carried out in the field of pelvic floor repair, which is expected to solve the current dilemma of pelvic floor mesh repair surgery (Figure 8G). Finally, in cluster 8, skin collagen implants, weight polypropylene mesh, marlex mesh, polymer dermis, periodic behavioral therapy, model, stress, strength, collagen, fibroblasts, and metabolism may be new research directions for pelvic floor surgery (Figure 8H).

## 4 Discussion

Pelvic floor defect repair surgery is an important sub direction of gynecological surgery (Giannini et al., 2019).The prediction study shows that the cumulative risk of women undergoing prolapse surgery at the age of 80 is 11% and the re-operation rate is estimated at 17%–30% (Denman et al., 2008; Maher et al., 2010; Wu et al., 2014). Even, the total number of POP operations will continue to increase by 50% in the next 40–50 years due to the continuous aging of the population (American College of O et al., 2019; Larouche et al., 2021). There are many types of pelvic floor surgery, the past 20 years have witnessed the initiation and important concerns of the application of pelvic floor mesh surgery (Wu et al., 2014; Mancuso et al., 2020). Since 2002, FDA has approved synthetic mesh for POP transvaginal mesh repair (van Geelen and Dwyer, 2013). At the initial stage, the preliminary application of pelvic floor mesh surgery showed a good application prospect because of its high anatomical success rate and low recurrence rate (D'Angelo et al., 2019). With a surge of mesh medical devices coming to the market, pelvic floor mesh surgery was rapidly popular. Over time, FDA noted an increase in reports of applications related to vacuum mesh, and issued two safety warnings in 2008 and 2011 (22). Finally, in the past 3 years, the competent authorities of the United States and parts of countries have successively issued bans on the sale and distribution of all transvaginal meshes (Hudson et al., 2020; Mao et al., 2021). In addition, advances in new fields such as biomaterials, tissue engineering and 3D printing have provided new prospects and directions for the development of safe and efficient individualized mesh surgery (Farmer et al., 2020; Farmer et al., 2021). In the face of policy changes and information explosion in this field, we conducted a bibliometric analysis to determine the current research hotspots, key points, keywords and trends of pelvic floor mesh surgery.

In the present study, we analyzed the relevant information of 2,550 articles, which were published in 349 journals by 2,294 institutions in 65 countries/regions, with 39,787 citations. Trend and annual analysis shows that there is a overall upward trend in the number of pelvic floor mesh-related publications and citations, although the number of publications fluctuated between 2013 and 2016. This phenomenon may be related to major events in the field of pelvic TVM surgery, especially the two safety warnings issued by the United States FDA (Clemons et al., 2013). It is worth noting that after some mesh products were banned and delisted in 2018, a relatively large number of pelvic floor mesh studies are still carried out internationally, reflecting the reflection and prospect of pelvic floor mesh surgery in the international community (Palmerola and Rosenblum, 2019; Sassani et al., 2020; Skorupska et al., 2020). The output of the United States, France, the United Kingdom, Italy, Australia and China ranks in the top six countries/regions of publications. The explanation for this may be that these countries are both countries with high incidence of POP and relatively developed science and technology (Huang et al., 2018). The institutional distribution is basically consistent with the national/regional distribution, the United States and France took the lead in terms of research Institution. In addition, countries/regions and institutions have close cooperation in this field, and cooperation between institutions is closer than that between countries/regions. Journals and co-cited journals analysis showed that the journals that published the most pelvic floor mesh surgery papers were International urogynecology journal (21.49%), Neurourology and urodynamics (4.902%), and Female pelvic medicine and reconstructive surgery (4.824%). International urogynecology journal (1,578 total citations) and Female pelvic medicine and reconstructive surgery (401 total citations) were frequently co-cited. Although the influencing factors and JCR divisions of these journals are not high, they are the main journals for pelvic floor disease research (Gupta et al., 2020).

The network analysis of 584 key words in the literature shows the research interest, and it is divided into 8 clusters: mechanism, type of prolapse, mesh complications, minimally invasive surgery, middle pelvic repair, anterior pelvic repair, quality of life, imaging examination, and bioengineering technology. These clusters mean that the research in these areas is popular and concerned in this field in the past 20 years. It is worth paying attention to that in cluster 1, it is related to the research on the molecular mechanism of POP. The current research mainly focuses on the metabolism and regulation of extracellular matrix, especially TGF-β, HOXA11, FBLN5, LOXL1, MMPs/TIMPs and other important target genes can enable us to better understand POP and provide new therapeutic targets for POP(Jameson et al., 2020; Deng et al., 2021; Li et al., 2021; Fang et al., 2022). In cluster 3, we have noticed that mesh complications was still a problem that needs high attention. On the one hand, we need to apply the standard terminology jointly developed by the International Urodynamic Association (IUGA)/International Continental Society (ICS) to register complications on a dedicated website (Haylen et al., 2011). On the other hand, we also need to be cautious about the indications of mesh, improve surgical techniques and attach importance to evidence-based evidence (Reid et al., 2021). In cluster 8, we found that tissue engineering has made excellent progress in POP treatment, especially scaffolding, seed cells, and growth factors may replace surgery to reconstruct natural tissues or use implants to treat POP(Yang et al., 2021; Radwan-Praglowska et al., 2020; Hennes et al., 2021). By keywords citation bursts analysis, the time-series showed that validation, lifetime risk, native tissue repair, tissue engineering and mesenchymal stem cells had appeared most recently. This result shows that clinical research is still the hot research topic of POP research, and prediction models and tissue engineering are research priority (Laursen et al., 2022; Oh et al., 2022; Zhang et al., 2022). In essence, overcoming mesh complications through increasing technical strategies is the core problem of pelvic floor reconstruction surgery. Another highlight of this study is that we also conduct in-depth manual classification of the network data of keywords, so as to overcome the shortcomings of machine classification strategies and further explore the research status of keywords. Manual classification divides keywords into the following 8 clusters: prolapse classification, risk factors, imaging progress, mesh surgery, mesh complications, quality of life, tissue engineering and new research directions. In addition to the similar results of machine reference analysis, four different new results are found based on the current research progress and the results of manual analysis. First, the fine diagnosis of three cavities and three levels is the basis for effective treatment (Petros, 2011; Chen et al., 2021); Second, imaging research may be one of the important directions to overcome mesh complications (Gavlin et al., 2020; Mahoney et al., 2020; Ram et al., 2021); Third, clinical research is an effective method to find answers to clinical questions, especially the clinical prediction model based on real world data (Barber and Maher, 2013; Weber LeBrun et al., 2018; Morcos et al., 2021); Finally, the new direction of tissue engineering technology and pelvic floor research is worthy of our expectation and further exploration (Hennes et al., 2021; Laursen et al., 2022).

To our knowledge, this is the first in-depth bibliometric analysis of pelvic floor mesh surgery, there are several limitations that need to be explained. First, the retrieval, download and analysis of this study from a single WoSCC database may lead to the loss of relevant important literature, which may lead to selective deviation of research conclusions. Second, the search deadline is 31 December 2021, excluding 2022 data, and relevant literature is still emerging in the database. Third, the bibliometric analysis carried out by machine algorithm does not carry out in-depth discussion on a single literature, and may not fully reflect all information. Last, international concerns about the indications for the use of mesh in pelvic floor reconstruction are growing rapidly, and bans in many countries and regions may have an important impact on relevant research. Nevertheless, based on limited data information and scientific methods, our research may help to intuitively understand the pelvic floor mesh surgery’s research hotspots and development trend.

## 5 Conclusion

In summary, to the best of our knowledge, this study is the first to use bibliometric methods to explain the current research status and global emerging trends of pelvic floor mesh surgery research in the past 20 years. This study shows that even though mesh complications have not been completely solved, international interest in pelvic floor mesh has always been strong. The United States is the country with the largest output, and England, Italy, Australia, Germany, France and China are the main participants, and close multidisciplinary collaborative networks can be found among institutions and among authors. The research focuses on the rational use of pelvic floor mesh and overcoming mesh complications, involving research hotspots in such areas as prolapse classification, risk factors, imaging progress, mesh surgery, mesh complications, quality of life, tissue engineering and new research directions.

## Data Availability

The original contributions presented in the study are included in the article/supplementary material, further inquiries can be directed to the corresponding authors.
